# A Dynamic Model of Hip Joint Biomechanics: The Contribution of Soft Tissues

**DOI:** 10.1155/2019/5804642

**Published:** 2019-06-04

**Authors:** Joseph F. Fetto

**Affiliations:** The Brooklyn Hospital Medical Center, Brooklyn, NY 11201, USA

## Abstract

Before recent advances in computer modeling technology, it has been nearly impossible to define the contribution of soft tissue structures when constructing models of the body, and in particular the lower extremity. For almost 100 years, the design and fixation of femoral components for total hip arthroplasty (THA), whether cemented or press fit, have been predicated on the Koch model of hip biomechanics. A more comprehensive model, which includes the dynamic contribution of soft tissues, has expanded the Koch's static model. This new model has led to a more complete representation of reality and has become the basis for the inclusion of a new stem design element (a lateral flare), a new concept of implant fixation (rest fit), and consequent significant increase in bone preservation and implant stability.

## 1. Introduction

It has been long recognized that soft tissues, such as ligaments and muscle-tendon units, are important stabilizing structures of articulations. However, the exact magnitude and character of their contribution have been the subject of much controversy. Historically, paradoxes have been recognized in the articulations of the lower extremity which have dramatized this issue. For example, if osteoarthritis (OA) is considered to be a “wear and tear” process, the incidence of OA in the joints of the lower extremity should be proportional to load/unit area (L/A) over time. Since the hip, knee, and ankle are exposed to an identical number of cycles of load and carry approximately the same body mass, the relative incidence of OA in each of these articulations, over time, should be proportional to their relative size. However, it has been universally recognized that the knee, the largest of these articulations, is the most frequently undergoing arthroplasty; and the ankle appears, unless its architecture has been disrupted, virtually immune to OA over a lifetime of use. Implicit in this reality is the conclusion that OA is more than a L/A phenomenon.

In its most basic form OA is the consequence of compromise of the integrity of the surface layer of the articular cartilage. This leads to a loss of the unique hydrostatic, frictionless nature of intact articular movement. Stability of an articulation protects an articular surface from excessive frictional wear. As such the incidence of OA will be inversely proportional to the stability of a given articulation, with stability being provided by a combination of geometry and soft tissue stabilizers crossing a given joint.

In this manner, it is predictable that the incidence of OA occurring in the geometrically unstable knee joint consisting of two large round condyles of the distal femur resting on a flat tibial plateau knee joint will be greater over time than that in the very geometrically stable ankle, where the talus is keystoned into the mortise of the ankle.

In addition to bony geometry, articulations are also stabilized by the soft tissues crossing that joint. For each plane of movement, these soft tissue structures can be subdivided into couples composed of a static (ligamentous) component and a dynamic (muscle-tendon) component. In this fashion it is apparent that the knee is much more dependent upon soft tissues for stability than is the ankle. It further explains why the relatively smaller ankle becomes so rapidly compromised and arthritic following unrepaired disruption of the syndesmosis ligament.

This importance of soft tissue stabilizers and their consequence for the loading of the lower extremity articulations have been of great consequence in the understanding and treatment of many conditions of the hip joint. In 1917, John Koch published his model of hip biomechanics [1]. It was to become accepted as the definitive model of the hip and lower extremity, for the next century. This model became the basis for the design of femoral implants employed in millions of hip arthroplasty surgeries performed around the world. Although intuitively attractive, this static model was plagued with many internal contractions and paradoxes.

It correctly assumed that the body's center of gravity (COG) was located in the midline of the body, 1 cm anterior to the first sacral segment. From this point the effect of gravity on the lower extremity was represented by a vertically oriented, downward directed vector. From these assumptions, it was proposed that during* unilateral stance* the weight of the body (B) would create both a compressive and varus load on points along the entire lower extremity. It further suggested that the magnitude of this varus deforming force acting at any point along the lower extremity could be calculated by multiplying the vector force created by gravity (body's weight) by its perpendicular medial displacement from that point along the lower limb (b), or B x b. It further hypothesized that this destabilizing torque must be counterbalanced by an equally strong valgus torque in order to maintain a stable equilibrium state during the gait cycle.

When Koch applied this analysis to the hip joint, he assumed the counterbalancing valgus torque would be supplied by isometric contraction of an abductor muscle (A), specifically the gluteus medius. He further assumed that the average length of the body's lever arm (b) from the center of the hip's rotation was approximately twice that of the length of the abductor's insertion on the lateral aspect of the greater trochanter to the center of hip rotation (a), b = 2a.

**Figure 1:**
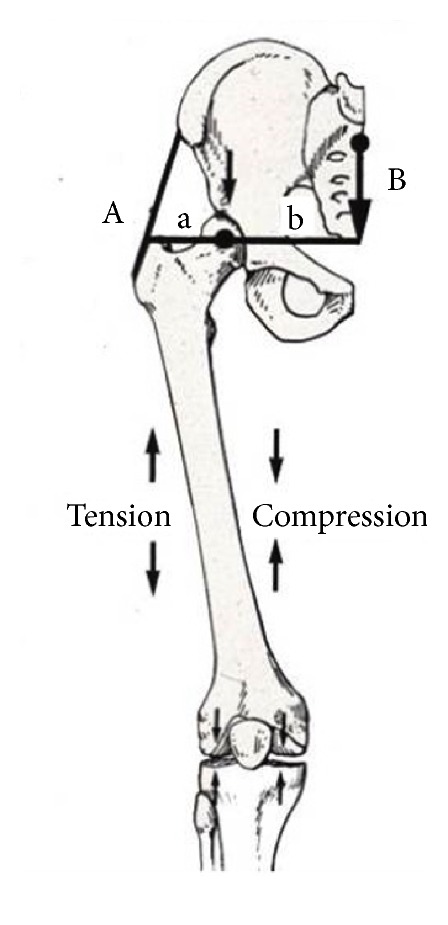
The Koch model of hip biomechanics (1) is static cadaveric model, (2) demonstrates his hypothesis of hip loading, and (3) requires the gluteus medius to exert a force twice that of the body's weight in order to maintain equilibrium during single stance.

From these assumptions, Koch wrote the equation for hip stability as B x b = A x a. And since b = 2a, he concluded that the gluteus medius must generate twice the weight of the body force, 2 x B, in order to maintain a stable equilibrium during unilateral stance. (Figure 1).

However, a more critical examination of Koch's assumptions must be undertaken before accepting his model as an accurate depiction of reality and in particular events occurring during gait.

## 2. History of Hip Biomechanics

**Figure 2:**
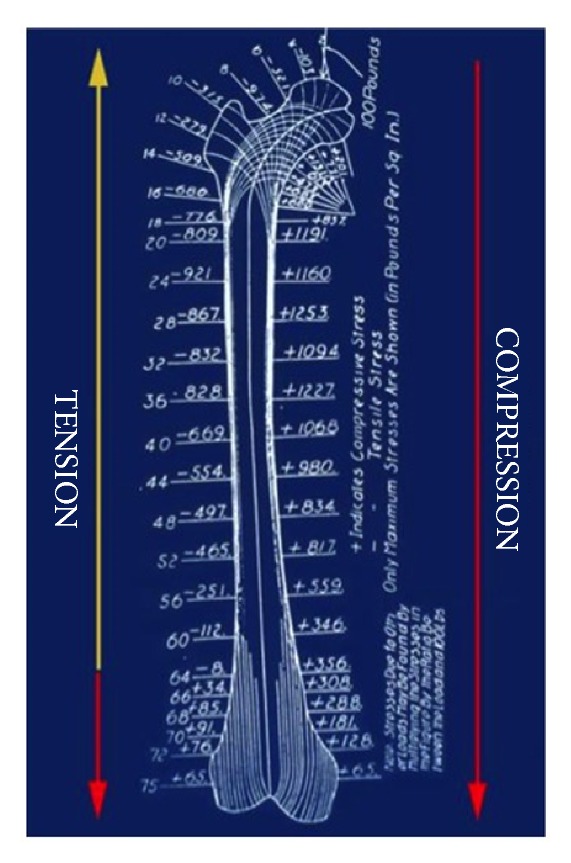
Koch's “quantification” of forces within the femur [1]. According to the Koch Model, during unilateral stance, (1) most of the lateral cortex experiences tensile loading and (2) the distal 1/3 of the lateral cortex and entire medial cortex experiences compression.

To begin with, Koch's model is static, unlike the conditions that occur during gait, where the body's center of gravity usually remains medial to the supporting foot in a dynamic equilibrium. Secondly, Koch limits the source of resistance to the body's varus load to a single structure, the gluteus medius. These two factors have dramatic consequences for conclusions drawn from the Koch model. The most important of them is the conclusion that, below the insertion point of the gluteus medius, the entire lateral aspect of the lower extremity experiences a tensile load. Koch went so far, in his original article, as to attempting to qualify the loads experienced by the femur during unilateral support. He used positive integers to represent compressive forces and negative integers to designate areas of tensile loading (Figure 2). Intuitively, it can be understood that he labeled the entire medial aspect of the femur as experiencing compressive loading. However, curiously he does not explain how the lateral femur experiences tension along its lateral proximal 2/3's but coverts to compression along the lateral distal 1/3 of its length. It may be assumed that Koch was attempting to keep his model consistent with the reality, and contemporary belief, that there is compression within the lateral compartment of the knee. It was believed, at that time, to be the explanation for the presence of a lateral meniscus within the knee's lateral compartment. An element intended to buttress against compressive loading.

The static model raised additional paradoxes. For example, at birth the neck-shaft angle of the femur is similar to that of a quadruped, approximately 160-165 degrees. As one assumes, in bipedal gait, by the age of four, the neck-shaft angle reduces to its final value of 130-135 degrees and remains so throughout the remainder of a lifetime. This has been explained as being the result of the upright posture placing a varus load on the young plastic bone. However it is interesting, that in spite of significant time spent in an upright posture and with increasing body mass, the femoral neck-shaft angle remains relatively unchanged throughout the remainder of growth and development of the femur.

It is further interesting to note that in neuromuscular conditions involving spasticity, the valgus deformity of the femoral neck is often corrected with a varus osteotomy. However, if insufficient soft tissue releases are performed at the time of the osteotomy, the valgus deformity will reoccur over time in spite of bipedal stance.

Another paradox of the Koch model occurs among lower extremity amputees. It has been found that below knee amputees (BKA) do not usually exhibit a positive Trendelenburg gait pattern. They only lose about 10% of their metabolic efficiency and with today's technological and material advancements can function at near normal levels of performance, while above knee amputees always exhibit a positive Trendelenburg gait pattern and lose 40-70% of their metabolic efficiency. Yet both the BKA and the AKA have intact abductor, gluteus medius, and musculature. The question is an obvious one. What is lost in an AKA that creates such a significant compromise of the gluteus medius' ability to provide stability against the varus load of the body during gait? (Table 1)

These clinical paradoxes suggest an explanation for why femoral components whose designs are based on the static, flawed, or at least incomplete Koch model of hip loading have provided unintended consequences. They have been plagued with outcomes which have included loss of proximal femoral bone mass termed “stress shielding”; diaphyseal hypertrophy; thigh pain; subsidence due to “poor bone quality”; loosening; and fracture on insertion.

**Figure 3:**
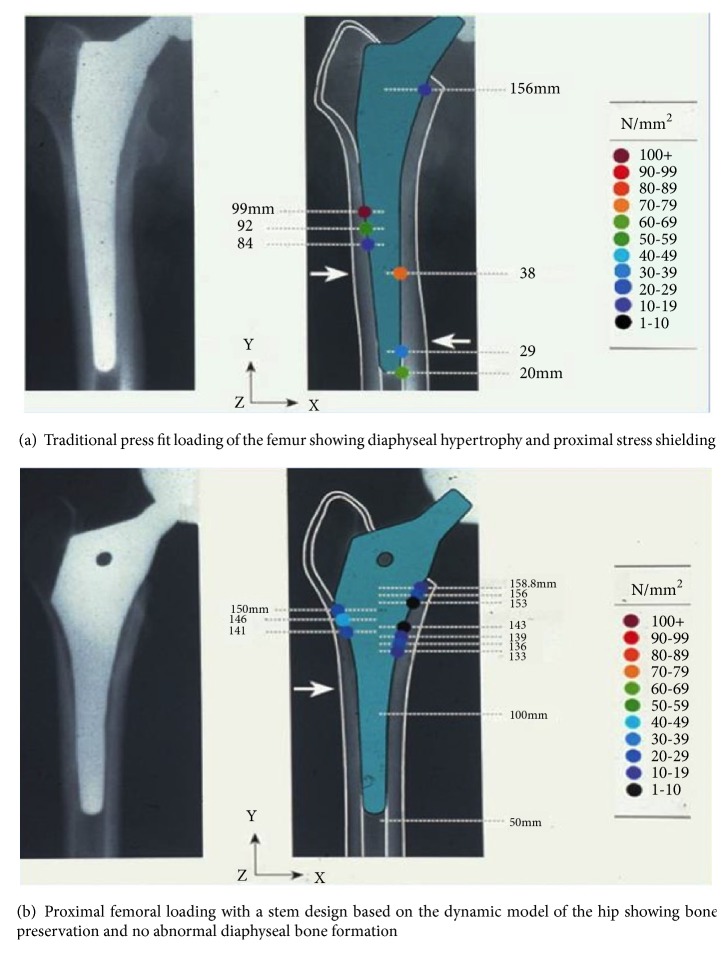
2-year stress distribution within the femur as a consequence of stem design.

Bone, as all connective tissues, responds to its environment. It hypertrophies and atrophies in response to demand. Also, the quality of bone reflects the type of load it experiences: cortical bone appears in areas of compression and cancellous bone in areas of tensile loading, i.e., apophyses and points of tendon attachment. It would appear that, rather than “reconstruct” damaged hip anatomy, femoral component designs and various methods for their fixation, based on the Koch model, have created nonphysiologic patterns of loading within the femur following hip replacement surgery (Figures 3(a) and 3(b)).

Over the past 150 years, there have been many theories proposed as models of hip biomechanics [2–7]. In efforts to avoid the paradoxes raised by the Koch model, each proposed the inclusion of additional structures to complement and supplement the action of the gluteus medius. However, due to a lack of the necessary means of investigation to prove their theory, all of these alternative models have been unable to displace the accepted model of Koch. This situation changed in the later 20th and early 21st century with the introduction of technological advances such as electromyograhic (EMG) studies, Finite Element Analysis (FEA), and radiologic techniques such as radio-stereotactic analysis (RSA) and DEXA analysis of bone density.

Electromyographic studies of Inman [8] directly challenged the conclusion of the Koch model. He demonstrated that the gluteus medius was most active just before and just after the midstance phase of gait. This implied that some additional factor was acting at midstance phase of gait to reduce the demand on the abductor musculature. The question was, How is this accomplished and what structure(s) is responsible?

## 3. Articular Cartilage

Unrelated was the observation that although the knee, hip, and ankle joint go through an equal number of load cycles throughout life and carry a similar load, it remains a fact that the smallest of these joints, the ankle, appears to be the most resistant to mechanical wear and development of osteoarthrosis requiring replacement. An explanation for this phenomenon can be deduced from an understanding of the ultrastructure of articular cartilage.

Articular cartilage is the soft covering at the end of bones and is present in all joints. It is the product of chondrocytes and is an avascular tissue, dependent upon the passive and active diffusion of nutrients and water through a porous surface layer. Chondrocytes produce two critical components of articular cartilage. They are collagen and proteoglycan. Collagen fibers are formed by long linear cross-linked triple helix molecules, specifically designed to resist tensile loading. It is paradoxical that they would be crucial to the integrity of articular cartilage, which is specifically intended to function in a compression environment. The key to the understanding of this paradox is the orientation of the collagen fibers within the cartilage cap. They are parallel at the surface forming a “skin” and dive from the surface into the hyaline cartilage to anchor themselves perpendicularly into the subchondral bone. As such they form an arcade in which the fibers have a specified orientation. Their orientation is such that as water is drawn into the cartilage tissue by the hydrophilic nature of the proteoglycan molecules, the surface layer of the cartilage structure is tethered to the subchondral bone by the collagen fibers, as it is pushed outward analogous to that of the surface of a stuffed pillow. The integrity of this structure then allows, when a compressive load is applied, water to exude from the cartilage to create a frictionless hydrostatic bearing between the surfaces of any joint. Thus, unless anything occurs to compromise the integrity of the hyaline cartilage surface layer, the cartilage cap is virtually immune to wear over time. From this description it is easy to understand how the demise of articular cartilage and resultant arthrosis can come about: proteolytic enzymatic action from bacteria of synovial inflammation; macrotrauma such as fracture; metabolic abnormalities affecting collagen or proteoglycan synthesis such as vitamin C deficiency and mucopolysaccharidoses; or most importantly microtrauma caused by excessive frictional wear. This last causative etiology is the direct result of joint instability.

Joint stability is a function of two factors: articular geometry and soft tissue integrity. In terms of geometry, it is easy to describe the three major joints of the lower extremity as being either inherently geometrically stable or unstable. The ankle, although the smallest of these three joints, has an extremely stable mortise structure with very limited degrees of freedom, particularly in the dorsiflexed position during weight bearing. As such it suffers minimal frictional wear throughout a lifetime unless the integrity of the mortise is compromised by either a fracture or more subtly disruption of the syndesmosis ligament. In either event, translational movement increases across the articular surfaces and demise of the ankle is rapid. With regard to soft tissues contributing to the stability of a joint, they can be divided into static and dynamic structures. Each plane of movement is stabilized by a couple comprised of a dynamic and a static entity. The dynamic structure is a muscle-tendon unit and the static stabilizer is a ligament. Muscles can adjust their length in response to demand. Ligaments however cannot. Hence since ligaments are critically important at extremes of motion and are lax at mid-range, all ligaments are composed of two parts: one taut in extension, the reciprocal part taut in flexion.

Because joint stability is critical to the preservation of the integrity and functionality of articular cartilage, and the knee joint is an inherently geometrically unstable articulation, it is easy therefore to understand and predict the knee's greater susceptibility to becoming arthritic over time.

## 4. The ITB Dynamic Tension Band Effect on the Hip Joint Biomechanics

Applying this type of analysis to the hip joint, it is possible to characterize the hip as being more geometrically stable than the knee but less so than the ankle. This is particularly true in the sagittal plane. In this plane the geometry of the hip provides no resistance against the varus deforming force which occurs during midstance phase of gait. The hip therefore must rely on a dynamic stabilizer such as the gluteus medius and some static stabilizer as well in order to maintain equilibrium at this phase of the gait cycle.

Cadaveric studies, in which a weight was suspended from the midline of the sacrum while soft tissue structures were divided into various combinations and permutations, demonstrated that the iliotibial band (ITB) is that static stabilizer of the hip against varus loads [9].

**Figure 4:**
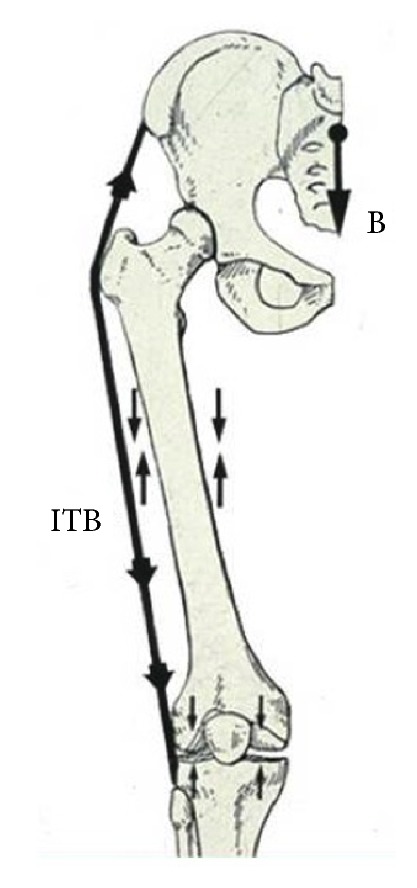
Compression band effect of the ITB. As a “tension band” lateral to the femur, the ITB neutralizes tensile loads and creates compression loading along the lateral aspect of the femur during unilateral stance phase of gait. This explains the presence and distribution of cortical bone in the lateral femur and is consistent with Wolff's Law.

From this observation, it was concluded that the ITB should be included in the analysis of hip biomechanics to produce a more complete, dynamic, and accurate model of hip stability. The inclusion of the ITB served to resolve many of the paradoxes raised by the previous static model. It resolved the seeming contradiction of the gluteus medius being less active at the midstance phase of gait. At that point of the gait cycle the ITB apparently serves as a tension band to relieve the metabolic demand and reduce electrical activity of the gluteus medius (Figure 4). It also provides an explanation for the poorer functioning of an AKA relative to that of a BKA, where loss of the distal attachment of the ITB in an AKA compromises the function of the ITB as a static stabilizer of the hip joint. It further gives rationale to the surgical technique of tenodesis of the ITB and lateral soft tissue structures to the distal femur in the performance of an above-the-knee amputation. This would be similar to the technique of wrapping the posterior calf musculature around the distal end of a below-the-knee amputation.

This more dynamic model, which includes the ITB as a tension band, provides an explanation for the presence of cortical bone along the lateral aspect of the femur as being a predictable consequence of compression loading (Figure 4). Extending this concept to the knee, it is also possible to predict the actual relative magnitudes of compressive loads in the medial and lateral compartments of that joint, which are 60% and 40%, respectively. Similarly, it explains, as Wolff's Law [9] predicts, the relative growth and dimensions of the medial and lateral femoral condyles being the consequence of the lateral stabilizing and tension band effect of the ITB, LCL, biceps femoris, and popliteus creating gradually increasing compressive loads across the distal femoral growth plate.

## 5. Femoral Component Design

Returning to femoral component design, it becomes possible, with this expansion of the Koch model to a dynamic model of the hip, to explain the shortcomings of component designs predicated on the 1917 model. Those previous designs treated the femur as a static element. Their placement and stability were analogous to that of a nail in a piece of wood, with the stability of the implant depending on a combination of friction and hoop-stress displacement. Their survival is totally dependent upon the quality of the host bone into which the component is being “pressed”. The risk was for subsidence with the choice of an undersized component, insertion into “poor quality bone”, or femoral fracture on insertion due to excessive force.

**Figure 5:**
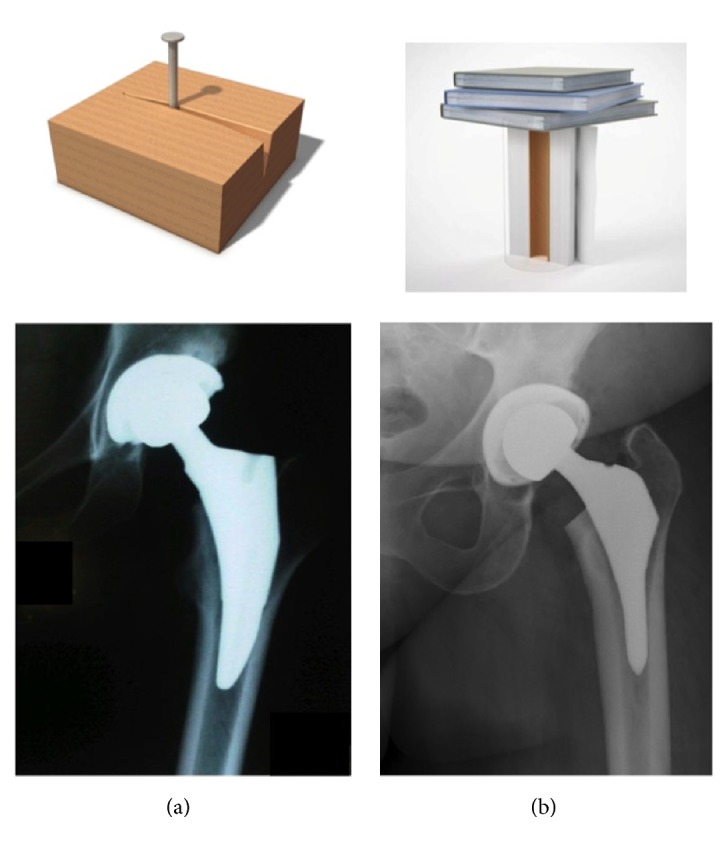
Press fit (a) vs rest fit (b).

In contrast to traditional means of achieving implant stability whether cemented or noncemented, the dynamic ITB model suggests an alternative method of achieving long term implant survival and superior outcomes for THR. The dynamic ITB model predicts that the lateral femur distal to the greater trochanter experiences compressive loading. Therefore it becomes reasonable to utilize the lateral endosteal surface of the proximal femur as an additional area to support the femoral component. In this way the entire circumference of the femur would be utilized as a pedestal upon which the body, as well as a femoral component, will be supported. The requirement for the design of a femoral component to achieve this “rest fit” would be to extend the lateral dimension of the component so as to engage the endosteal surface of the femur in the region of Gruen zone 1. This lateral expansion, or “lateral flare”, would create an internal collar which would provide the means to transfer load to the entire proximal perimeter of the femur (Gruen zones 1 and 7). As such the component could rest on the entire femur, distributing load both medially and laterally in a more physiologic fashion (Figure 5). This design concept predicts the means to preserve proximal bone stock and avoid transfer of nonphysiologic loading into more distal Gruen zones 2,3,4,5, and 6. It also negates the belief that a long stem design element is required to achieve fixation. Rather the stem portion of a component could simply serve as an alignment device and not a load bearing structure. It would also predict that stem length may be safely reduced without compromising stability or longevity of the construct.

## 6. Merit of the Lateral Flare and Rest Fit Fixation

The validity of these predictions was evaluated by several means: FEA, in vitro laboratory testing, and finally prospective DEXA and subsidence clinical studies. These published results have shown that, unlike prior stem designs which demonstrate stress shielding, diaphyseal hypertrophy, thigh pain, subsidence, and occasional fracture on insertion, the “lateral flare” stem design has minimized these adverse outcomes. Lateral flare stems have demonstrated preservation of >95% bone stock in proximal Gruen zones 1 and 7; less than 0.5 mm subsidence; no fracture on insertion of the stem when employing a “rest fit” rather than press fit insertion technique; and no thigh pain [10–13].

## 7. Conclusion

In conclusion, over the past 30 years the application of recent technological advances through laboratory and clinical investigations has led to an expansion of the static Koch model of 1917 to create a more accurate and complete understanding of hip biomechanics. This has produced a more valid dynamic model of the hip. The consequences of this expansion has led to a better explanation of the growth and development of the femur, the functional differences between BKA and AKA patients, suggestions for improved surgical techniques in various clinical situations, and the addition of an important design element to femoral components, specifically the “lateral flare”. More importantly this new understanding of hip biomechanics has advanced femoral fixation from a static “nail in a piece of wood” press fit technique to an improved means of femoral fixation termed a “rest fit”, which has demonstrated improved outcomes in total hip arthroplasty.

## Figures and Tables

**Table 1:** Importance of an intact ITB to amputee function.

	Iliotibial band	Gluteus medius	Trendelenburg sign	Energy expenditure
BKA	Intact	+	−	−10%

AKA	Transected	+	+	−40 ~−70%
